# A systematic review of mental health care workers' constructions about culturally and linguistically diverse people

**DOI:** 10.1371/journal.pone.0200662

**Published:** 2018-07-19

**Authors:** Tinashe Dune, Peter Caputi, Beverly Walker

**Affiliations:** 1 Faculty of Social Science, School of Psychology, University of Wollongong, Wollongong, New South Wales, Australia; 2 School of Science and Health & Translational Health Research Institute, Western Sydney University, Penrith, New South Wales, Australia; USC Keck School of Medicine, Institute for Global Health, UNITED STATES

## Abstract

A systematic review of research published in English was conducted across seven electronic databases in psychology, health and social sciences. The aim was to ascertain the nature of mental health care workers’ constructions about culturally and linguistically diverse individuals in order to facilitate provision of culturally appropriate service delivery and multicultural training. The constructs and perspectives of 5,870 mental health workers with regards to minority populations are represented across the 38 studies included. Key themes comprised: *Aetiology of Constructions*; *Content of Constructions*, *Factors that Influence Constructions*; *Implications for Cultural Competence*, *Implications for the Therapeutic Alliance*, *Recommendations for Training*, *Recommendations for Practice* and *Recommendations for Research*. The therapeutic alliance was most at risk when practitioners displayed low levels of cultural competency and high levels of racial and ethnic blindness. The changing and increasingly multicultural context within most countries means that mental health systems and workers need to prepare for an increasing range of culturally and linguistically diverse clients in need of support. Recommendations are explored for training, practice and research.

## Introduction

Migration within an international context has increased exponentially especially in recent decades. Migrants are often required to learn a new language, cultural, ethnic customs and/or religious ideologies [1]. They may also contend with changed roles as well as becoming a minority in their adoptive countries. Being a minority can also apply to indigenous populations, like those in countries where the colonisers never left. Minority populations are sometimes characterised as culturally and linguistically diverse (CALD)–a term intended to acknowledge those who identify as being of a different cultural, ethnic, religious, linguistic and/or racial background than the dominant group in a particular country [2]. The term CALD is acknowledged to be problematic and its use widely debated. While such debate is beyond the purview of this paper the term CALD was used in light of its broad scope in relation to minority populations. Further reflection on the term CALD and other alternatives is included within the Discussion section of this review.

The process of adapting to fundamental changes of identity, beliefs, behaviours and/or values between cultures can exacerbate or trigger mental health concerns, depending on the host context in which change occurs (e.g., hospitable versus inhospitable host societies) [3].

Not only do migrants need to acculturate in order to adapt to their new environments but the host society must also adapt to accommodate an increasingly multicultural society [4, 5]. This is particularly imperative for health care systems and their practitioners, who must address a wide range of individual and group health and wellbeing needs [6]. To adequately support CALD clients, health care workers are expected to espouse principles of cultural competency that support an effective therapeutic alliance [7, 8]. However, success in this respect requires wide-ranging acceptance and integration of diversity across systems, institutions and personal practice. Without this type of commitment, the health and wellbeing of CALD people cannot be adequately supported.

To explore these issues a systematic literature review was conducted to better understand (i) how mental health care workers construct CALD people or clients, (ii) what might be the implications of these constructions for cultural competence and the therapeutic alliance and (iii) their significance for both CALD people and mental health care workers. This paper therefore draws on Kelly’s (1955) Personal Construct Theory whereby constructions of an individual’s reality are based on how the individual construes social events, people, and/or themselves (i.e., constructs) and the processes by which they engage in construal–Kelly’s ‘Fundamental Postulate’.

### Mental health workers’ constructions about CALD people and the role of cultural competency

Little is known regarding mental health workers’ constructions about CALD people. Current understandings of their constructions are implied from the extensively documented negative health outcomes and experiences of CALD people across a range of health care settings (see for example [9]). In general, the core of these experiences are related to health systems and workers harbouring constructions of health and wellbeing based on Euro/ethno-centric frameworks [10]. As such, mental health systems and practitioners often demonstrate resistance to incorporating alternative constructions of health and wellbeing into service provision [11, 12]. To circumvent negative outcomes researchers advocate for increased cultural competency [2].

“Knowledge, conviction and capacity for action at an individual and organisational level” are embedded within the concept of cultural competence [13]. Eisenbruch [14] also emphasises the skill-based notion of competence that encompasses the system (including health workers) no less than the patients or clients. As such, cultural competency is defined as:

A set of congruent behaviours, attitudes and policies that come together in a system, agency or among professionals and enable that system, agency or those professionals to work effectively in cross-cultural situations [15].

Building health care workers’ abilities to become self-aware and to reflect on their constructions about others is an important aspect of cultural competency training [2]. The primary goal of this training is to improve the health and wellbeing outcomes of clients by improving the practitioner-client therapeutic alliance. Effective engagement in cultural competency is designed to promote the development and maintenance of a positive therapeutic alliance between practitioners and their clients [2].

### The therapeutic alliance and mental health outcomes

The therapeutic alliance is broadly defined as “the collaborative and affective bond between therapist and patient… [and] is an essential element of the therapeutic process” [16]. With its origins in early psychoanalytic theories (e.g., [17–19]), the concept is now a staple of contemporary understandings and evaluations of the therapeutic process. It is well understood that the therapeutic alliance is related to clients’ mental health engagement and outcomes–for better or for worse [16]

### Exploring constructions about CALD people

There is a long history of research on minority clients’ perceptions and experiences of health care, practitioner cultural competency and the therapeutic alliance. A systematic review exploring the barriers and enablers to ethnic minority patient engagement with health services found 54 articles from around the world [20]. Three levels of barriers were identified: patient level, provider level and system level. Barriers at all levels included beliefs, attitudes, skills, behaviours and systems that valued Western constructions of health and wellbeing as well as the ‘other’ [20]. This aligns with a systems approach to cultural competence and the need for all levels of society to ‘acculturate’ to the realities of multiculturalism. There is also growing research on health practitioners’ cross-cultural attitudes, beliefs, values, awareness, responsiveness and their cultural competency (see for example [4, 21–25]), indicating that the therapeutic alliance is most at risk when practitioners demonstrate low levels of cultural competency and high levels of racial and ethnic blindness. This can result in the perception that people of culturally and linguistically diverse backgrounds should be treated equally to all others, irrespective of the impact of their diversity on health. Prevailing Euro/ethnocentric attitudes and values embedded within all aspects of social, health, economic, and political structures that reinforced racism, discrimination, prejudice and exclusion [6] reduced cultural competency and limited therapeutic alliance.

The above studies focused on a broad range of health practitioners, including doctors, nurses and allied health practitioners. Relatively few studies addressed similar themes with mental health practitioners and mental health care systems, despite researchers such as Chao [26] arguing that similar concerns apply. A paucity of research collating and presenting a collective perspective on the aetiology of mental health practitioner constructions, the content of those constructions, the factors which influence or mediate constructions and the impact of constructions on cultural competence and the therapeutic alliance is available.

To address these queries a systematic literature review was conducted exploring: how do mental health care workers construct CALD people or clients and what might be the implications of these constructions on cultural competence and the therapeutic alliance?

## Methods

In line with Preferred Reporting Items for Systematic Reviews and Meta-Analyses (PRISMA) guidelines [27], a systematic review was conducted to identify research that investigated mental health care workers’ constructions about CALD people (see **S1 Table**). The implications of these constructions on cultural competence and the therapeutic alliance were also considered.

### Search strategy

The focus was seven electronic databases in psychology, health and social sciences: PsycINFO, ProQuest Central, PubMed, ScienceDirect, Scopus, CINAHL Plus and Infomit. The search for published literature in English was undertaken during April and November 2016. Following Mengesha et al. [6], a step-by-step search strategy was employed.

A preliminary search of ProQuest Central was undertaken by the first author with the goal of identifying the key words contained in study titles and abstracts and ascertaining index terms used to describe articles. Pertinent key words were discussed, expanded and refined with the other authors. A second search, using all identified keywords, was conducted across the seven databases indicated. Finally, the reference lists of all included literature were examined for additional studies. Details of the search strategy, including the search terms and combinations, are summarised in Table 1.

**Table 1:** Inclusion/Exclusion criteria and keywords.

Parameters	Inclusion	Exclusion	Key words/steps
Location	International	None	N/A
Language	Written in English	Other languages	Select for English only
Time	Any	None	N/A
Population	Literature which include mental health care workers	Literature which do focus on mental health care workers	((Abstract) Psychologist OR Psychiatrist OR Mental health nurse OR Mental health worker OR Counsellor OR Social worker OR Psychotherapist OR Therapist)
Phenomena/Target	Studies concerned with the participants’ constructions, beliefs, attitudes, views and perceptions of CALD clients	Not concerned with the participants’ constructions, beliefs, attitudes, views and perceptions of CALD clients	AND ((Abstract) Construct OR Beliefs OR Values OR Attitudes OR Perceptions OR Stereotypes) AND ((Abstract) Visible minority OR Visual minority OR Culturally and Linguistically Diverse OR Non-White OR ethnic minority OR racial minority OR linguistic minority OR language minority OR English as Second Language OR Language other than English OR Language Background other than English OR English as an Additional Language or Dialect)
Study/literature type	Published primary research including qualitative, quantitative and mixed method designs	Published literature which DO NOT include qualitative, quantitative and mixed methods of data collection and analysis	N/A

### Data synthesis

The review analysed literature by a thematic approach developed by Thomas and Harden [28] that extracted, synthesised, analysed and interpreted the findings of the included literature. Three steps were followed: 1) line by line coding of the results, discussion and conclusion sections of the primary studies; 2) development of descriptive themes; and 3) generation of analytical themes towards a synthesised presentation of results. The first author completed a preliminary synthesis of primary data followed by a review and disagreement resolution by all authors.

### Quality assessment

In order to assess the quality of the quantitative papers included in this systematic review we used the Effective Public Health Practice Project’s *Quality Assessment Tool (QAT) for Quantitative Studies* [29]. The QAT was used to appraise 27 of the 38 studies included, those that employed quantitative or mixed methods approaches to collecting, analysing and interpreting the data. Studies that used only qualitative methods were not evaluated using the QAT. The first author assessed the quality of studies with the second and third authors reviewing the article assessments.

## Results

From the 583 potentially relevant articles identified, 38 articles were included in the systematic review (**S1 Fig**).

### Sample

The characteristics of each study are summarised in Table 2. The perspectives of 5,870 mental health workers with regards to CALD people were synthesised and are presented under Theme 1 below. Most studies (77%) did not focus on a specific CALD population. CALD groups discussed in the remaining 23% included: Eastern Europeans, Asians and/or Asian-Americans, Blacks and/or African Americans, Latinos and Mexicans, Arabs or those from the Middle East, Jews, refugees and migrants. Fifteen papers included the perspectives of mental health graduate students. There were 11 papers focused on mental health workers in a range of occupations (e.g., occupational therapists, social workers, special education teachers, nurses and general practitioners). There was a low presence of research with clinical psychologists or psychotherapists (6), psychiatrists (2), speech pathologists/therapists (2) and school counsellors (2). The vast majority of research was conducted in the United States (28), with the remainder being in Canada (3), Australia (3), Japan (1), Tanzania (1), the United Kingdom (1) and Germany (1). The total number of studies included may not add up to the number of papers with a particular focus area, sample or country as some studies included more than one of the above in their analyses.

**Table 2:** Characteristics of 38 studies included in the systematic review.

Reference No.	Literature Type	Type of Mental Health Worker/s	CALD group/ Country	Mental Health Care/Setting	Theoretical approach	Sample size	Design/data collection	Analytical approach
[30]	Journal Article	Counsellors	CALD/Canada	N/A	N/A	181	Qualitative: Critical Incidents	Content Analysis
[31]	Journal Article	Therapists	Ethnic minorities/USA	Family Therapy	Experiential family therapy	6	Qualitative	N/A
[32]	Journal Article	Counsellors	Ethnic minorities/ Tanzania	Counselling university students	N/A	1	Autoethnography	N/A
[33]	Journal Article	Psychotherapists	Mexican-American, “Negroes”, Japanese-Americans, Chinese-Americans and Jews/USA	Therapeutic relationship	N/A	16	Qualitative: interviews	N/A
[34]	Journal Article	Counselling graduate students	CALD/USA	Multicultural Training	N/A	84	Quantitative: surveys	Multilevel modelling
[35]	Journal Article	Counsellors	CALD/USA	Multicultural competency	N/A	338	Quantitative: surveys	Hierarchical multiple regression
[26]	Journal Article	School counsellors	CALD/USA	Multicultural competence	N/A	259	Quantitative: surveys	ANOVA, regression analysis
[36]	Journal Article	School counsellor trainees	CALD/USA	Multicultural counselling competence	N/A	99	Quantitative: surveys	MANOVA, hierarchical multiple regression
[37]	Journal Article	School counsellors	Immigrant students/USA	Counselling immigrant students in schools	N/A	139	Multicultural case conceptualisation, Quantitative: surveys	Hierarchical multiple regression
[38]	Journal Article	GPs, nurses, psychologists, occupational therapists & social workers	CALD/ Australia	Acute adult inpatient	N/A	53	Qualitative: focus groups	Thematic analysis
[39]	Journal Article	Counselling students	CALD/USA	Multicultural training	N/A	516	Quantitative: surveys	Multiple regression, ANOVA, hierarchical regression
[40]	Journal Article	Social workers and psychologists	Racial minorities and women/USA	N/A	N/A	705	Quantitative: surveys	Descriptive statistics
[41]	Journal Article	White counselling and clinical psychology trainees	Racial minorities/USA	N/A	N/A	177	Quantitative: surveys	Multivariate multiple regression
[42]	Journal Article	Health and human service professionals	Refugees/ Australia	Refugee-specific services	N/A	22	Qualitative: semi-structured interviews	Thematic analysis
[43]	Journal Article	Special education teachers	CALD students/ USA	Special education	N/A	17	Quantitative: online survey	N/A
[44]	Journal Article	Counsellor	CALD/USA	Multicultural counselling training	N/A	1	Auto-ethnography	N/A
[45]	Journal Article	Psychology trainees	Refugees/ Canada	Culturally grounded supervision of Psychotherapy practica	N/A	9	Mixed Method: Quantitative (pre/post survey) Qualitative (journal entries) Auto-ethnography	Exploratory analyses, t-tests and thematic analysis
[46]	Journal Article	Psychiatrists, doctors, psychiatric social workers and psychologists, nurses, medical students	Black and ethnic minorities/UK	Psychiatric diagnosis	N/A	339	Clinical vignette, Quantitative: questionnaire	N/A
[47]	Journal Article	School speech-therapists	CALD students/ USA	Schools	N/A	9	Qualitative: semi-structured interviews	Thematic analysis
[48]	Journal Article	Counselling students	Minority clients/USA	N/A	N/A	120	Quantitative: survey	Multiple regression analyses
[49]	Journal Article	European American mental health practitioners	CALD/USA	Multicultural counselling competencies	N/A	412	Quantitative: survey	MANOVA
[50]	Journal Article	Social Worker	CALD social worker/ Australia	Child Protection	Auto-ethnography	1	Auto-ethnography	N/A
[51]	Journal Article	Private Psychotherapists	Cross-cultural interactions/ Germany	Outpatient mental health care service	N/A	485	Quantitative: survey	Chi-squared, t-tests, one-way ANOVA, Mann-Whitney U test, factor analysis
[52]	Journal Article	Multicultural therapy trainees	CALD/USA	Multicultural therapy training	N/A	38	Quantitative: survey and Guided Inquiry	MANOVA, frequency and percent ratings
[53]	Journal Article	Mental health workers, applied psychology students	CALD/USA	Multicultural counselling competencies	N/A	130	Quantitative: survey	MANOVA
[54]	Journal Article	Counselling graduate students	Refugee, Immigrant/USA	Mental health outreach program	N/A	12	Qualitative: Interviews	Thematic analysis
[55]	Journal Article	Psychiatrist	Japanese/ Canada	Transcultural psychiatry	N/A	1	Autoethnography	N/A
[56]	Journal Article	White counselling graduate students	CALD/USA	Multicultural counselling competencies	N/A	128	Quantitative: survey	Hierarchical regression
[57]	Journal Article	White counselling graduate students	CALD/USA	Multicultural training	N/A	116	Quantitative: survey	Factor analysis, 2 x 2 mixed model ANOVA, primary ANOVA, ANCOVA
[58]	Journal Article	Counsellors	CALD/USA	Multicultural counselling competencies	N/A	220	Quantitative: survey	Descriptive analysis, MANOVA & ANOVA
[59]	Journal Article	Counsellors	CALD clients/USA	Multicultural counselling competencies	N/A	220	Quantitative: survey	Descriptive analysis, MANOVA & ANOVA
[60]	Journal Article	Counsellors, speech pathologists, special education teachers	CALD/ USA	Schools	Non-experimental descriptive design	75	Mixed Methods: survey	Descriptive analysis & thematic analysis
[61]	Journal Article	Counselling trainees	CALD/USA	Mentoring of ESL students	Consensual qualitative research	67	Qualitative: self-reflection process notes Quantitative: survey	Thematic analysis, ANOVA, Pearson correlations, Mean Difference
[62]	Book Chapter	Doctoral counsellor trainees	Immigrant school children/USA	A middle school English as a Second Language Program/ Multicultural Counselling Training	N/A	16	Qualitative: trainees' process notes and Quantitative: survey	Thematic analysis, Chronbach’s alpha, Pearson correlation coefficient
[63]	Journal Article	Counselling trainees	CALD/USA	Multicultural Counselling Training	Critical Incidents Technique	124	Qualitative and Quantitative: Critical incidents	Content analysis, chi-square
[64]	Journal Article	White counselling trainees	CALD/USA	Multicultural counselling competencies	N/A	311	Quantitative: survey	Descriptive analysis, MANOVA & ANOVA
[65]	Journal Article	Regular and special educators	Multicultural students/USA	(Special) Education	N/A	403	Quantitative: survey	N/A
[66]	Journal Article	Counselling psychology graduate students	CALD/USA	Ethnic bias in counselling	N/A	20	Illusory correlation paradigm & questionnaire	Independent group t-test, paired t-test

### Research foci and theoretical approach

The studies primarily focused on evaluating practitioner attitudes, skills and behaviour related to (multi)cultural competency (20) or the impact of cultural competency training (8). Other research directly or indirectly explored the impact of these measures on health care outcomes (8), patient/client diagnosis (4) and the therapeutic alliance (6) within mental health settings. All studies implicitly or explicitly aimed to make recommendations about (multi)cultural competency training and practice as well as means for improving the therapeutic alliance and CALD patient/client outcomes.

Only five papers explicitly indicated the use of a theoretical approach to guide the research. Among those that did were auto-ethnography, experiential family therapy, non-experiential descriptive design, consensual qualitative research and critical incidents.

### Research design and methodology

Quantitative methodology using surveys were the most common data collection strategy. Eleven used qualitative approaches, with interviews and focus groups being most common. Nine used a mixed methods approach. Given the emphasis on quantitative methods, a variety of statistical analyses were applied including descriptive statistics, multilevel modelling, multiple regression, MANOVA, ANOVA and hierarchical regression. Qualitative studies undertook thematic or content analyses. Nine studies did not specify the analytical approach used (see Table 2).

### Study quality

As shown in Table 3 the QAT included six major assessment criteria: a) selection bias, b) study design, c) confounders, d) blinding, e) data collection methods and f) withdrawals and drop-outs. Fifteen specific quality assessment sub-criteria where weighted according to the guidelines described for use of the QAT [29]. Global scores for each major criterion, based on the responses to the sub-criteria, provided an overall indication of a study’s quality level: Strong = 1, Moderate = 2 and Weak = 3. Although study quality varied significantly across the 15 quality assessment sub-criteria all 27 studies assessed where found to be Weak given that they attracted more than two Weak scores across the six major criteria indicated above.

**Table 3:** Quality assessment results.

QAT major criteria	Selection Bias			Study Design					Confounders				Blinding			Data Collection Methods			Withdrawals and dropouts			Global QAT Rating
Reference No.	Representative Sample	% ppl agreed to participate	Overall SB rating	Type	Randomised—Y/N	Randomisation described?	Appropriate Method?	Overall SD rating	Diff. b/w groups?	Type	% controlled confounders	Overall Confounders rating	Blinded researchers?	Blinded Participants?	Overall Blinding rating	Valid data collection tools?	Reliable data collection tools?	Overall DC rating	Withdrawls reported? w/ reasons?	% part. who completed intervention	Overall WD rating	
[34]	2	5	3	3	N	n/a	n/a	2	N	n/a	n/a	3	2	2	3	1	1	1	2	n/a	3	3
[35]	2	5	3	7	N	n/a	n/a	3	3	n/a	n/a	3	2	2	3	1	1	1	4	n/a	3	3
[26]	1	3	2	7	N	n/a	n/a	3	N	n/a	n/a	3	2	2	3	1	1	1	4	3	3	3
[36]	2	5	3	7	N	n/a	n/a	3	N	n/a	n/a	3	2	2	3	1	1	1	4	4	3	3
[37]	1	2	2	7	Y	n/a	n/a	3	N	n/a	n/a	3	2	2	3	1	1	1	1	2	2	3
[39]	1	2	2	7	N	n/a	n/a	3	N	n/a	n/a	3	2	2	3	1	1	1	1	2	2	3
[40]	1	3	2	7	Y	n/a	n/a	3	N	n/a	n/a	3	2	2	3	1	1	1	1	3	2	3
[41]	2	5	3	7	N	n/a	n/a	3	N	n/a	n/a	3	2	2	3	1	1	1	4	4	3	3
[43]	1	1	1	7	N	n/a	n/a	3	N	n/a	n/a	3	2	2	3	3	3	3	1	4	3	3
[45]	3	5	3	5	N	n/a	n/a	3	N	n/a	n/a	3	2	2	3	1	1	1	4	4	3	3
[46]	1	1	1	7	N	n/a	n/a	3	N	n/a	n/a	3	2	2	3	3	3	3	1	1	1	3
[48]	2	5	2	7	N	n/a	n/a	3	N	n/a	n/a	3	2	2	3	1	1	1	4	4	3	3
[49]	1	3	2	7	N	n/a	n/a	3	N	n/a	n/a	3	2	2	3	1	1	1	3	4	3	3
[51]	1	1	1	7	N	n/a	n/a	3	N	n/a	n/a	3	2	2	3	3	3	3	1	1	1	3
[52]	2	4	3	5	N	n/a	n/a	3	N	n/a	n/a	3	2	2	3	1	1	1	4	4	3	3
[53]	2	4	3	7	N	n/a	n/a	3	N	n/a	n/a	3	2	2	3	1	1	1	1	4	2	3
[56]	1	2	2	7	N	n/a	n/a	3	N	n/a	n/a	3	2	2	3	1	1	1	1	2	2	3
[57]	2	1	2	3	Y	n/a	n/a	2	Y	training	Y	2	2	2	3	1	1	1	2	3	3	3
[58]	1	2	2	7	N	n/a	n/a	3	N	n/a	n/a	3	2	2	3	1	1	1	1	2	2	3
[59]	1	2	2	7	N	n/a	n/a	3	N	n/a	n/a	3	2	2	3	1	1	1	1	4	2	3
[60]	4	4	3	7	N	n/a	n/a	3	Y	profession	4	3	2	2	3	1	1	1	4	4	3	3
[61]	2	4	3	7	N	n/a	n/a	3	N	n/a	n/a	3	2	2	3	1	1	1	4	4	3	3
[62]	2	4	3	7	N	n/a	n/a	3	N	n/a	n/a	3	2	2	3	1	1	1	4	4	3	3
[63]	4	4	3	7	N	n/a	n/a	3	N	n/a	n/a	3	2	2	3	3	3	3	4	4	3	3
[64]	4	4	3	7	N	n/a	n/a	3	N	n/a	n/a	3	2	2	3	1	1	1	4	4	3	3
[65]	2	3	3	7	N	n/a	n/a	3	N	n/a	n/a	3	2	2	3	3	3	3	1	3	2	3
[66]	4	5	3	7	N	n/a	n/a	3	N	n/a	n/a	3	2	2	3	3	3	3	4	4	3	3

### Major findings

Following line-by-line coding of the extracted results and discussion sections from individual studies, three major themes with subthemes emerged: *Construing CALD People*, *Impact of Constructions* and *Recommendations*. In the interest of brevity and readability major findings are accompanied by only some example citations. The terms ‘workers’ or ‘practitioners’ is used in place of ‘mental health care workers’ for ease of reading.

#### Theme 1: Construing CALD people

Aetiology: Practitioners constructions about CALD people originated from social, cultural, political, religious and economic hierarchies that valued ethnocentric norms and values [30, 31, 46, 59, 65]. With the majority of research originating from Western countries, Eurocentric norms and values prioritised Whiteness and manifestations of White supremacy (e.g., racism and institutionalised discrimination). In these papers [41, 52, 53, 57, 67], CALD people were constructed as social problems and were perceived as causes of societal instability within a range of cultural, political, religious and economic arenas. The ethno/Eurocentric aetiology and hierarchies produced both challenging and contradictory constructions about CALD people.

Content: All studies indicated that constructions about CALD people manifested in worker attitudes, beliefs, assumptions and biases as well as stereotypes and prejudices. These constructions, especially prior to cultural competency training, were noted as barriers to CALD people’s health outcomes and were a major focus of training interventions. Even so, constructions about CALD people sat on a spectrum from negative (e.g., migrants are economic drains), to neutral (e.g., racial/ethnic/colour blindness), to positive (e.g., they add social and economic wealth to their adoptive societies). While some studies did not articulate specific constructions (most were simply indicated to be ‘attitudes’ or ‘stereotypes’) the majority of perspectives could be grouped into construct sets. These constructs, their place on this spectrum and the literature that discussed these constructions are presented in Table 4, with an ‘x’ indicating presence of construction. Although some constructions were negative or problematic prior to cultural competency training, changes to such constructions were dependant on a range of moderators and mediators within, and external to, workers’ control.

**Table 4:** Constructions about CALD people in each of the 38 studies.

Constructions	1	2	3	4	5	6	7	8	9	10	11	12	13	14	15	16	17	18	19	20	21	22	23	24	25	26	27	28	29	30	31	32	33	34	35	36	37	38
**Negative**																																						
CALD people have backward values/beliefs	x					x		x					x				x		x	x		x	x	x		x	x	x					x		x			x
CALD clients do not know what is best for them	x							x									x		x	x		x	x	x		x		x					x		x			
CALD people’s behaviours are limited to stereotyped caricatures		x			x	x		x					x							x		x		x		x		x							x			x
CALD people are economic and social drains				x		x							x							x		x		x			x	x							x			
CALD people display more antisocial behaviours than non-CALD people																		x		x		x		x		x	x	x					x		x			x
CALD people are poor and uneducated				x															x	x		x		x		x		x					x		x			x
CALD people are defensive/ sensitive	x	x		x													x	x	x	x		x		x				x							x			
**Neutral**																																						
CALD people see things differently than non-CALD people	x					x	x	x					x		x	x	x		x	x	x	x	x	x	x	x	x	x	x	x	x		x	x	x	x	x	x
CALD people are quite different to non-CALD people		x			x	x		x	x	x			x	x			x		x	x	x	x	x	x	x			x	x	x	x		x		x	x	x	
CALD people are compared against the dominant group		x			x	x	x	x	x				x	x	x	x	x		x	x	x	x	x	x	x		x	x	x	x	x	x	x	x	x	x	x	
Not all CALD people only want to interact with people of their background			x					x	x				x	x		x	x		x	x	x	x	x	x	x	x		x	x	x	x			x		x	x	
**Positive**																																						
CALD people are not simply stereotypes		x	x				x	x	x	x			x	x	x	x	x	x	x		x	x		x		x			x	x			x	x	x			
Not all CALD people and groups are the same			x				x	x	x	x		x	x	x		x	x	x	x		x	x		x		x			x	x			x		x			
CALD people experience prejudice across generations		x					x	x	x	x		x	x	x		x	x		x		x	x		x		x	x		x	x			x	x	x			
CALD people are important sources of information and knowledge							x	x	x	x		x	x	x	x	x	x	x	x		x	x	x	x		x			x	x		x	x	x	x			
CALD people are important and necessary in society								x	x	x		x		x		x			x		x	x		x		x			x	x		x	x		x			
CALD people are grateful for the mental health support they receive										x				x	x	x			x			x				x	x			x								

Factors that influence constructions about CALD people: In addition to cultural competency training, major influences included workers’ sex, ethnocultural background and exposure to CALD people both within and external to counselling practice. The importance of practitioner characteristics were examined through a range of quantitative measures related to cultural competency, ethnic/racial/colour-blindness, White Racial Identity as well as qualitative explorations of critical incidents and autoethnographic accounts. For the most part no gender/sex differences were statistically significant with regards to constructions about CALD people, cultural competence, racial identity or colour-blind attitudes. In a few studies, female mental health workers’ espoused more positive constructions about CALD people than males and perceived themselves to be more culturally competent than their male counterparts [39]. Generally, workers’ who identified as being CALD had higher levels of cultural competency prior to multicultural training [26, 59, 64] while other studies demonstrated no such differences [63]. These results became relatively equal after multicultural training, with non-CALD workers’ improving marginally more than their CALD counterparts [64]. Most research indicated that workers who had social and/or clinical experience with CALD people had higher levels of cultural competence, less ethnic/racial/colour-blind attitudes and/or more advanced White Racial Identity frameworks. “Advanced” White Racial Identity refers to Whites who operate primarily from the later stages of this racial development framework. These stages include: Contact, Disintegration, Reintegration, Pseudo-independence, and Autonomy. In the final stage, for example, “Whites internalize a positive racial identity by no longer imposing arbitrary racial definitions on others and by displaying an intellectual and emotional appreciation of racial differences and similarities” (Constantine, 2002, p. 164). These factors further influenced practitioners’ multicultural knowledge, awareness, relationship and counselling skills–with higher scores in each area being linked to positive constructions about CALD people and higher cultural competence.

#### Theme 2: Implications of constructions

Implications for cultural competency: Constructions about CALD people were intimately linked to practitioner levels of cultural competence. This was explicitly evident in studies using the Multicultural Counselling Inventory (MCI) [34, 35, 39, 45, 49, 56, 58, 59, 61, 68]. Within each subscale (i.e., Skills, Knowledge, Awareness and Relationship), items that revealed respondents’ constructions (e.g., stereotypes, beliefs and values) were assessed in relation to mental health support tasks, skills and knowledge. Across several studies using the MCI and/or other competence scales, workers’ perception of their skills- and knowledge-based competence was generally higher than their awareness- and relationship-based competencies. Positive correlations were also present between negative or neutral/positive constructions and limited or heightened cultural competency, respectively. This is corroborated by other scales (e.g., White Racial Identity or Color-blind Racial Attitudes Scale) analysed independently and alongside the MCI as well as other methodologies (e.g., critical incidents and qualitative methods).

Although cultural competency training is designed with these associations in mind, practitioners with negative constructions were more likely to resist changing their constructions or were more dissatisfied with such training [36, 52, 53, 61, 62, 64, 69]. Conversely, those with more neutral and positive constructions found that cultural competency training increased their interest in, and comfort with cultural diversity [44, 45, 63] as well as their desire to engage with those from cultures unlike their own [32, 50, 55]. As such, workers constructions are important to development of the therapeutic alliance.

Implications for the therapeutic alliance: Mental health workers’ felt that their constructions could result in minor limitations to the therapeutic alliance [38, 42, 46]. Limitations include the inability to bridge cultural differences [30], micro-aggressions [41], value conflicts [44] or racial/ethnic/colour-blindness [35]. Even so, practitioners perceived themselves to have appropriate constructions and therefore appropriate levels of cultural competence as well as good engagement with clients/CALD people [38, 42, 46]. Notably, before cultural competency training, workers were often oblivious of the impact of some of their constructions on the therapeutic alliance and client outcomes [70]. This was demonstrated in studies that assessed worker constructions, competencies and engagement with clients’ pre and post training [45], particularly those including assessments of MHCWs racial/ethnic/colour-blindness [26, 34] and racial identity [41]. All the studies acknowledged that competency gaps could be addressed in practitioner training, practice and research.

#### Theme 3: Recommendations

Training: Further developments in training that reflect and accommodate the dynamic nature of culture and society were advocated in all studies. Key recommendations included the infusion of cultural competence in all aspects of practitioner training [30] in addition to stand alone units of study that tie all areas of learning together [34]. Further, cultural competency training should consistently focus on the role and impact of culture and diversity on the development and/or maintenance of practitioners’ constructions about CALD people. Training could move away from a focus on building awareness, knowledge or skills related to culture [52, 53], towards developing a habit of reflexivity in counselling practice [37, 42, 50, 55]. Oandasan and Reeves [71] reflexivity refers to:

“self and group reflective exercises, within safe learning environments, [where] students may begin to develop the reflective skills necessary for developing an appreciation and understanding of each other’s roles, their unique backgrounds and the professional perspectives on clinical decision making that ensures each profession is distinctive […]. Reflection can only occur if opportunities are provided […] that expose students to issues that they must grapple with”.

In doing so, learners should be supported “to explore and resolve possible racist [colour-blind] and intolerant attitudes and to make these efforts an ongoing feature of their continuing growth and self-exploration” [37]. In saying that, training must also respond to learner diversity, with cultural competency programs often focusing on the experiences of non-CALD people’s level of awareness, knowledge, skills and engagement, leaving these potentially bored and disengaged [26]. According to Chao (26) such individuals’ lived experience provides them with first hand exposure and engagement with cultural diversity, with current training doing little to improve their capacity for cultural competency.

Given the importance of the lived experience, most studies suggested that exposure to, and engagement with, people from culturally and linguistically diverse backgrounds through placement and cross-cultural supervision benefited all workers both during and post-study [45]. Greater exposure and engagement with CALD people manifests in more positive constructions and improved cultural competence [48]. This aspect of training is intimately linked with practitioner ability to support the therapeutic alliance. Trainees who only receive knowledge, awareness and skill-based training do poorly on the Relationship sub-scale of the MCI because they do not know how to apply what they have learnt to actual human beings [56]. Exposure and engagement is therefore an important opportunity for trainees to apply theory and become comfortable with being uncomfortable [44], breakdown resistance to cultural competency training [61, 72] and to effectively work through it [69].

Studies that collected data on continuing education found that the majority of their sample had not engaged in CALD-specific training since becoming a practitioner, with others (particularly those who had been practicing for over a decade) not having received any such training during their preparatory education [30]. The literature indicated that regular participation in continuing education, with a specific focus on CALD people, is required to keep up with changing and increasingly multicultural societies [39, 40, 45]. This could be supported by increased representation of cultural diversity amongst mental health students, faculty, placement experiences and supervisors [48].

Practice: To avoid a one-size-fits-all approach [38] applying the principles of cultural competence via culturally appropriate interventions is suggested [69]. This strategy reinforces the aims of training for cultural awareness and reflexivity [41]. Resources that focus on value conflicts in cross-cultural settings, and how to manage them, are necessary [55], as well as those that provide some guidance on cultural expectations or protocols within major ethnic groups [51]. The importance of interpreters in the provision of accessible and responsive mental health care was also mentioned [50]. In particular, practitioners needed to know how to access them and utilise their skill set [38]. In situations where practitioners worked with a large number of clients from a particular CALD group, the literature encouraged them to learn some of their clients’ language, to make home visits and to attend cultural events [47]. Services would also benefit from a diverse workforce [48] and actively supporting the inclusion of diverse clients in their practice. Practitioners are therefore encouraged to engage family members, where appropriate, as this may be of great importance to collectivist cultural groups [50]. Doing so also acknowledges clients and their families as knowers or experts on their own experiences and health needs [26, 35, 36, 47, 60]. This however may require more time than currently budgeted for counselling and educating CALD clients as compared to some non-CALD clients [53, 54, 65]. These inclusions in practice, amongst many other factors, are important institutional, organisational and systemic precursors to access and utilisation of mental health care services for CALD people [51, 55, 66].

Research: Future research can focus on the processes through which cultural competency training transfers into reflexivity, awareness, knowledge and skills and then into effective practice with CALD clients [30, 65, 66]. As such, differences in how various training experiences (e.g., didactic and experiential) influence constructions about CALD people requires further investigation [36, 73]. More information is also needed about trainees who are resistant, ambivalent or non-receptive to the principles of cultural competence and the means for supporting the therapeutic alliance [63]. This information would allow for the development and delivery of effective pedagogy as well as learning environments with a cultural ambiance conducive to attitudinal and behavioural change [39].

Further investigation of worker demographics can help to explore their influence on cultural competence and the therapeutic alliance [36, 47, 68]. A better understanding of the cultural competence and constructions about CALD people held by supervisors can reveal what trainees are taught ‘on-the-job’ about the therapeutic alliance and how to manage it [34, 56, 64]. Differences between experienced/practicing workers versus trainees could also be explored within varying global contexts, using a range of methodologies [52, 53].

With regards to research methods, a variety of theories and data collection techniques were advocated in order to provide a multidimensional understanding of slippery concepts like ‘constructions about CALD people’ and ‘CALD clients’ needs’ within the ‘therapeutic alliance’. Recognition was given to the difficulty of their assessment due to the likelihood of participants responding in socially desirable ways and reliance on self-report data [68]. Ways to mediate and/or account for these confounders of valid data were welcomed [52, 53]. The representativeness of the research samples were noted by authors as a limitation, with more research needed to develop a strong evidence base for training and practice [57]. Authors suggested that research move away from a deficit-model of CALD people, which may reinforce them as being “problems” that workers have to “deal” with, but are not sure how to. Instead a strengths-based model where CALD people are engaged in research to develop and challenge workers was proposed [32, 47, 50, 51, 55].

## Discussion

A systematic review of 38 studies published in English was conducted to ascertain the nature of mental health care workers’ constructions about culturally and linguistically diverse individuals in order to facilitate provision of culturally appropriate service delivery and multicultural training. The findings demonstrate that constructions about CALD people are intimately linked to workers’ ability to engage in ways that are culturally competent and supportive of an effective therapeutic alliance. This review has also highlighted that little is known about the explicit constructions that workers hold regarding CALD people as well as the views held outside of the United States. While some constructs were easy to identify, others were simply discussed as negative or positive ‘values’ or ‘biases’ or ‘stereotypes’, with a limited indication of their content–especially regarding negative constructs. This perhaps reinforces the insidious nature of negative constructions about CALD people which are not explicitly or fully articulated, but pervasive in all areas of society–making them hard to pinpoint and address [11, 74].

Even so, constructions were for the most part positive, particularly after multicultural competency training. While training improved competency for the majority of workers involved in such programs [61, 62, 72], there remain practitioners, across all helping professions, who reject or resist the premise of cultural competency training [2]. This is concerning given the positive benefits of such professional development. However, it may not be cultural competency training itself that some practitioners take issue with, but instead the way in which it is taught and the relatively limited gains which they perceive can come from it. For instance, CALD practitioners may already have the fundamental background knowledge required to be culturally competent. Consequently, their cultural competency scores start off higher than non-CALD practitioners and then level out post training. Given the limited post-training gains experienced by this group, cultural competence training may be perceived as boring, resulting in disengagement. For these practitioners the basic level at which some training occurs may not capitalise on the insights and experience that CALD workers already have.

While further research is warranted to explore differences and similarities with regards to constructions about CALD people, cultural competency and the therapeutic alliance, such explorations can be problematic and fraught with methodological, theoretical and conceptual difficulties. For instance, when trying to extract or identify such constructions we are reminded that not all CALD people are the same and therefore may not be construed similarity. Even the term ‘CALD’ fuels much debate as it lacks meaning for many scholars [75]–after all isn’t everyone a CALD person? As such, research can benefit for a more explicit and unapologetic delineation of the groups who are being discussed–which several of the included studies had done (e.g., focusing on Black Americans, Asians and/or Latinos). Given that the majority of studies discussed Whiteness, not simply as a colour but as the foundation for social/cultural/economic/political/religious systems, the term non-White may be appropriate for exploring several minority groups at once and acknowledging the role of Anglo/European culture on minority people [10, 11, 76]. Although Whiteness, as a societal system, is increasingly a global phenomenon these terms may not be useful in contexts where the dominant culture is not White (e.g., Japan).

The recommendations presented are therefore very important–with training as a necessary starting point [2, 77] and an understanding that a ‘one-size-fits-all’ approach is ill-advised. In an era of educational entitlement within tertiary settings [77], the ways in which cultural competency is taught needs reform to ‘reach’ and re/educate practitioners from a range of backgrounds and levels of ‘experience’–whether clinical or otherwise. Such reform would benefit from the development of evidence-based cultural competency training programs relevant to specific national contexts. This may include a focus on developing worker engagement and understanding of local (and international) CALD people through experiential learning and opportunities to apply theoretical knowledge. However, accreditation processes and institutional bureaucracy often limit the speed at which this can occur.

### Limitations

While this review presented a comprehensive synthesis of published research a potential limitation is that literature like unpublished masters and doctoral theses were excluded from the search. Even so Vickers and Smith [78] noted that after a review of the Cochrane Library, only one of 878 systematic reviews included data from theses that could have fundamentally altered the conclusions of those reviews. Nevertheless, Vickers and Smith [78] noted that the benefits of including dissertations in systematic reviews are minimal as they rarely influence the conclusions, whilst retrieving and analysing unpublished dissertations involves considerable time and effort.

This study is further limited by the disproportionate number of papers conducted in the US. As such the views presented are limited to those of a particular sociocultural, political and economic setting in which multiculturalism has been a long standing discussion resulting from America’s extensive history of migration and the aftermath of that history for many ethnic groups. Second, this means that the experiences and views of mental health care workers in other contexts, which have very different (and some with much shorter) histories of migration, are not heard, or drown in those from the USA. Whilst this may simply indicate that such conversations are in their infancy or adolescence (e.g., like in Australia) the lack of perspectives from other geographic regions limits insights for both the therapeutic alliance and cultural competency in relation to minority populations in those countries (e.g., Indigenous Australians). Third, given that only studies published in English were included bias exists when trying to make inferences about people from non-English speaking backgrounds. For instance, countries like France, Italy, Greece and Spain (to name a few) receive large numbers of migrants who are faced with integrating into a new sociocultural environment. Perspectives from these contexts would enrich understandings of mental health care workers’ constructions about CALD people. As such, more research that can inform locally relevant training and practice models in other languages than English and from regions outside of the US is required.

## Conclusion

Multiculturalism within an international context has increased exponentially, especially in the past two decades. While some CALD people arrive to their adoptive countries with existing mental health concerns, others develop such concerns following migration. To assist these clients in managing their mental health needs this systematic review reinforces that mental health care workers who construe CALD people in line with principles of cultural competency are better able to support a robust therapeutic alliance. This synthesis of existing evidence concerning mental health care workers’ constructions about CALD people reiterates that the provision of culturally-appropriate services are contingent on the cultural competency training delivered to mental health trainees. This review has found that the therapeutic alliance between minority clients and mental health care workers is most at risk when practitioners have negative constructions about CALD people that are correlated with low levels of cultural competency.

Within our multicultural global context the issue of cultural competency, or the lack thereof, requires urgent attention. While this does not mean that individuals and ethnocultural groups cannot retain their identities and beliefs, it *does* mean that:

We must prepare ourselves for a future that has arrived. We must educate our children to be global citizens who can live with all the paradoxes of identity, who can change while retaining a core sense of self. [79]

The changing and increasingly multicultural context of all nations means that mental health systems and workers need to be prepared for the range of clients in need of support. As the included literature notes, such a feat requires commitment to developing dynamic and responsive training, research and practice models.

## Supporting information

S1 Table PRISMA 2009 checklist. (DOC)

S1 Fig Article selection process. (DOCX)
