# Supplementary material for: A systematic review of mental health care workers' constructions about culturally and linguistically diverse people
Source: PLoS One. 2018 Jul 19;13(7):e0200662. doi: 10.1371/journal.pone.0200662 (PMC6053145; doi:10.1371/journal.pone.0200662)
Supplement: S1 Fig — (DOCX) [file pone.0200662.s002.docx]

Total number of literature identified from the seven databases (n=583)

Screened by title (n=409)

Excluded (n=174) articles because of duplicates

Excluded (n=295) because not relevant by title

Screened by abstract (n=114)

Full text evaluation (n=36)

Excluded (n=74) because not relevant by abstract

References from included literature screened by title and abstract (n=12)

Excluded (n=10) because not relevant by participants or subject

38 items incl. peer-reviewed articles (n=36) and book chapters (n=2) were included in qualitative synthesis

**S1 Fig. Article Selection Process**
